# Genetic diversity and immunogenicity of the merozoite surface protein 1 C-terminal 19-kDa fragment of *Plasmodium ovale* imported from Africa into China

**DOI:** 10.1186/s13071-021-05086-6

**Published:** 2021-11-24

**Authors:** Qinwen Xu, Sihong Liu, Kokouvi Kassegne, Bo Yang, Jiachen Lu, Yifan Sun, Wenli Zhong, Miaosa Zhang, Yaobao Liu, Guoding Zhu, Jun Cao, Yang Cheng

**Affiliations:** 1 Laboratory of Pathogen Infection and Immunity, Department of Public Health and Preventive Medicine, Wuxi School of Medicine, Jiangnan University, Wuxi, Jiangsu People’s Republic of China; 2 Key Laboratory of National Health and Family Planning Commission on Parasitic Disease Control and Prevention, Jiangsu Provincial Key Laboratory on Parasite and Vector Control Technology, Jiangsu Institute of Parasite Diseases, Wuxi, 214064 Jiangsu People’s Republic of China; 3 School of Global Health, Chinese Center for Tropical Diseases Research, Shanghai Jiao Tong University School of Medicine, Shanghai, 200025 People’s Republic of China

**Keywords:** *Plasmodium ovale*, Merozoite surface protein 1, Conservation, Immunogenicity

## Abstract

**Background:**

Merozoite surface protein 1 (MSP1) plays an essential role in erythrocyte invasion by malaria parasites. The C-terminal 19-kDa region of MSP1 has long been considered one of the major candidate antigens for a malaria blood-stage vaccine against *Plasmodium falciparum*. However, there is limited information on the C-terminal 19-kDa region of *Plasmodium ovale* MSP1 (PoMSP1_19_). This study aims to analyze the genetic diversity and immunogenicity of PoMSP1_19_.

**Methods:**

A total of 37 clinical *Plasmodium ovale* isolates including *Plasmodium ovale curtisi* and *Plasmodium ovale wallikeri* imported from Africa into China and collected during the period 2012–2016 were used. Genomic DNA was used to amplify *P. ovale curtisi* (poc) *msp1*_*19*_* (pocmsp1*_*19*_) and *P. ovale wallikeri* (pow) *msp1*_*19*_ (*powmsp1*_*19*_) genes by polymerase chain reaction. The genetic diversity of *pomsp1*_*19*_ was analyzed using the GeneDoc version 6 programs. Recombinant PoMSP1_19_ (rPoMSP1_19_)-glutathione S-transferase (GST) proteins were expressed in an *Escherichia coli* expression system and analyzed by western blot. Immune responses in BALB/c mice immunized with rPoMSP1_19_-GST were determined using enzyme-linked immunosorbent assay. In addition, antigen-specific T cell responses were assessed by lymphocyte proliferation assays. A total of 49 serum samples from healthy individuals and individuals infected with *P. ovale* were used for the evaluation of natural immune responses by using protein microarrays.

**Results:**

Sequences of *pomsp1*_*19*_ were found to be thoroughly conserved in all the clinical isolates. rPoMSP1_19_ proteins were efficiently expressed and purified as ~ 37-kDa proteins. High antibody responses in mice immunized with rPoMSP1_19_-GST were observed. rPoMSP1_19_-GST induced high avidity indexes, with an average of 92.57% and 85.32% for rPocMSP1_19_ and rPowMSP1_19_, respectively. Cross-reactivity between rPocMSP1_19_ and rPowMSP1_19_ was observed. Cellular immune responses to rPocMSP1_19_ (69.51%) and rPowMSP1_19_ (52.17%) induced in rPocMSP1_19_- and rPowMSP1_19_-immunized mice were found in the splenocyte proliferation assays. The sensitivity and specificity of rPoMSP1_19_-GST proteins for the detection of natural immune responses in patients infected with *P. ovale* were 89.96% and 75%, respectively.

**Conclusions:**

This study revealed highly conserved gene sequences of *pomsp1*_*19*_. In addition, naturally acquired humoral immune responses against rPoMSP1 were observed in *P. ovale* infections, and high immunogenicity of rPoMSP1_19_ in mice was also identified. These instructive findings should encourage further testing of PoMSP1_19_ for rational vaccine design.

**Graphical abstract:**

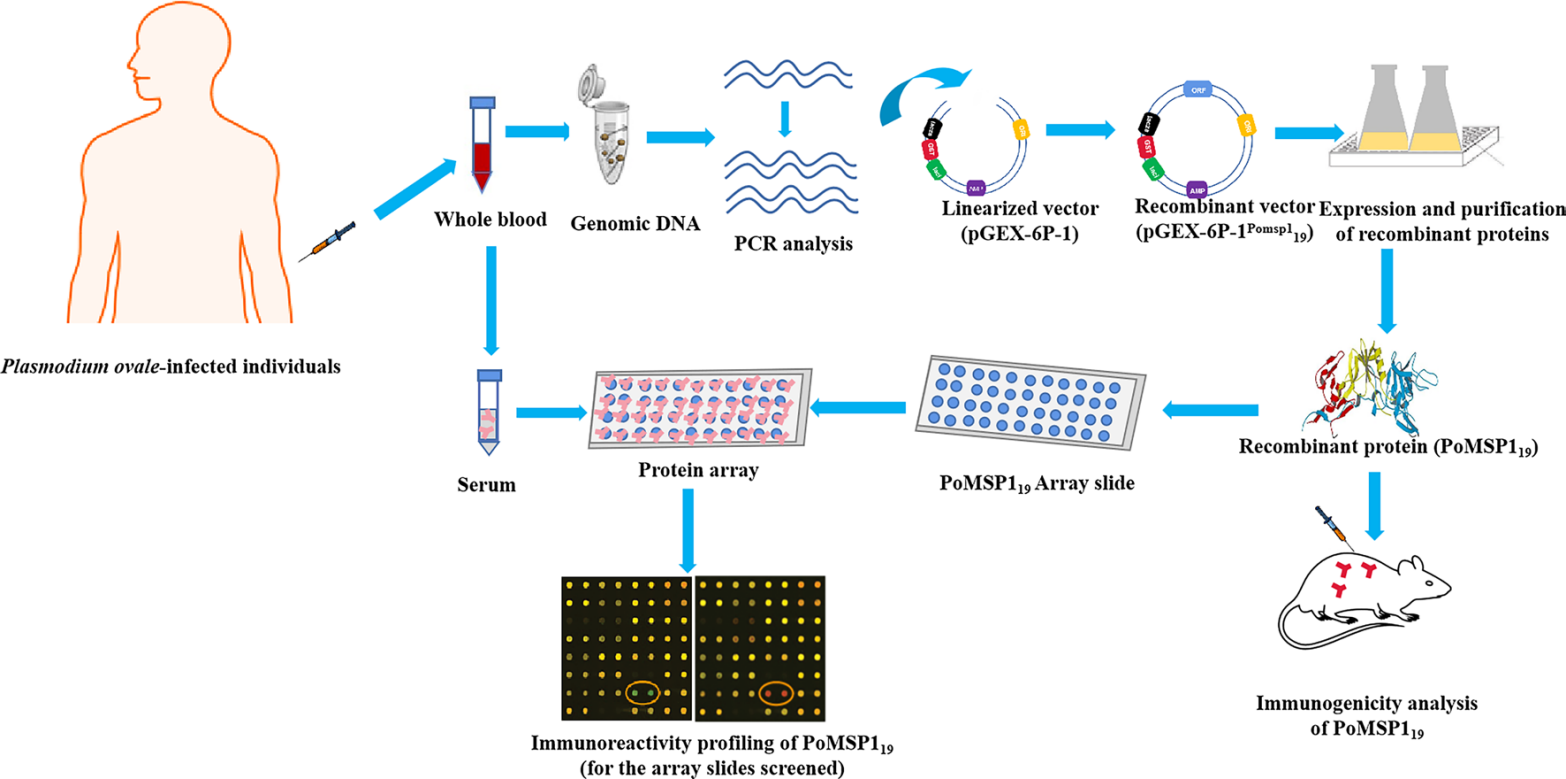

**Supplementary Information:**

The online version contains supplementary material available at 10.1186/s13071-021-05086-6.

## Background

Malaria is one of the most severe infectious diseases that threaten human health. According to the World Health Organization, there were 229 million clinical cases of malaria and 490,000 deaths from this disease in 2019 [1]. *Plasmodium ovale* is one of the five species of *Plasmodium* that regularly infect humans, and accounts for 0.5–10.5% of all malaria cases [2]. It is geographically distributed in sub-Saharan Africa and the Western Pacific Region and is classified into two subspecies: *Plasmodium ovale curtisi* and *Plasmodium ovale wallikeri* [2, 3]. *Plasmodium ovale* has a similar morphology and life cycle to *Plasmodium vivax* [2]. The prevalence of *P. ovale* malaria is underestimated because of the low density of these parasites in infected subjects, mild clinical symptoms (e.g. fever), and mixed infections with other species of *Plasmodium* [2, 4, 5]. However, *P. ovale* infection can evolve into severe malaria with severe anemia and may become fatal, especially in areas where malaria is endemic [6, 7]. Although there have been impressive achievements in research for *P. falciparum* malaria vaccines [8–10], no widely effective vaccine exists for *P. ovale* malaria. China has eliminated malaria within its borders [11], and was very recently certified as officially free of malaria by the World Health Organization. However, as a consequence of economic growth and deepening of the global trade the number of cases of malaria imported into China, including cases of *P. ovale* infection, increased in recent years [12–14].

During the invasion of human erythrocytes by malaria parasites, the merozoite surface proteins (MSPs) (including MSP1) are exposed to the host immune system [15]. Moreover, antibodies targeting MSP1 have been observed in individuals from malaria-endemic areas and have been shown to confer immunity [16–18]. The molecular weight of MSP1 is approximately 200 kDa [19]. The MSP1 protein undergoes two proteolytic cleavage steps during invasion of the erythrocytes. In the first step, MSP1 is cleaved into four polypeptides (with molecular weights of 83 kDa, 30 kDa, 38 kDa, and 42 kDa). Subsequently, the 42-kDa fragment is cleaved into 33-kDa (MSP1_33_) and 19-kDa (MSP1_19_) fragments; the latter remains attached to the merozoite surface and enters the erythrocytes [20–22]. Several studies have identified limited polymorphism in the C-terminal 19-kDa region of both *P. falciparum* MSP1 (PfMSP1_19_) [23–25] and *P. vivax* MSP1 (PvMSP1_19_) [26, 27]. Genetic conservation within vaccine candidate antigens is advantageous for vaccine development as it helps to reduce antigenic escape [27, 28]. Moreover, antibodies against PfMSP1_19_ can prevent the invasion of merozoites into erythrocytes [29]. Antibodies to PfMSP1_19_ have been associated with protection from malaria in pregnant women, infants and older children naturally infected with *P. falciparum* [30–32]. These instructive findings imply that MSP1_19_ is a promising candidate antigen for a blood-stage vaccine.

Genetic polymorphisms of MSP1 in clinical *P. ovale* isolates from Thailand were analyzed and showed low sequence diversity [33]. However, there is a paucity of information on PoMSP1_19_. In the present study, sequences of *pomsp1*_*19*_ from clinical *P. ovale curtisi* and *P. ovale wallikeri* isolates from patients with *P. ovale* malaria imported into China from Africa were investigated. In addition, the immunogenicity of PoMSP1_19_ was assessed in mice, and the levels of immune responses against PoMSP1_19_ assessed in serum samples of patients infected with *P. ovale*.

## Methods

### Collection of samples

Blood samples of febrile patients who had returned from work in malaria-endemic areas of sub-Saharan Africa during the period 2012–2016, and who were confirmed for *P. ovale curtisi* or *P. ovale wallikeri* infection by polymerase chain reaction (PCR) tests [13], were obtained from local hospitals in Jiangsu Province, China [34, 35]. Genomic DNA was extracted from the blood samples for PCR amplification of *pomsp1*_*19*_ genes. In addition, serum samples of the *P. ovale*-infected patients (*n* = 29) who had returned from Africa and those of healthy individuals (*n* = 20) from China were also obtained from the local hospitals of Jiangsu Province.

### PCR amplification and sequencing of ***pomsp1***_***19***_

A total of 37 clinical *P. ovale* isolates (*P. ovale curtisi*, *n* = 20; *P. ovale wallikeri*, *n* = 17) were collected for PCR amplification (Additional file 1: Table S1). Sequences of *P. ovale curtisi* (poc) *msp1* (*pocmsp1*) (KC137343) and *P. ovale wallikeri* (pow) *msp1* (*powmsp1*) (KC137341) genes from the GenBank database of the National Centre for Biotechnology Information were used as reference sequences [33, 34]. The 258-bp sequences of *pomsp1*_*19*_ were identified via matching with similar sequences, as previously reported [36, 37], and were amplified by nested PCR. The first-round primers were as follows: *pomsp1*_*19*_ forward (5′-AGT AAG GAA AAA GAT TTG ACA A-3′) and *pomsp1*_*19*_ reverse (5′-AAG TAA GTT AAA TAG GAT GAT-3′). The primers for the nested PCR were as follows: *pomsp1*_*19*_ forward (5'-ATG GGA TCT AAA CAT AAA TGT-3') and *pomsp1*_*19*_ reverse (5'-GAA AAC ACC TTC GAA GAA TGG-3'). All the amplification reactions had the same reaction conditions, as follows: 98 °C for 3 min, followed by 35 cycles of 98 °C for 10 s, 45 °C for 1 min, and 72 °C for 1 min, and final extension at 72 °C for 5 min. PCR products were electrophoresed on 1.2% agarose gels for visualization under an ultraviolet transilluminator (ChemiDoc MP; Bio-Rad), then purified and sequenced by Genewiz (Suzhou, China). In addition, PCR-amplified fragments were also cloned into the pUC57 vector and sequenced by Genewiz using the following primers: M13F, 5′-TGT AAA ACG ACG GCC AGT-3′; M13R, 5′-CAG GAA ACA GCT ATG AC-3′. To evaluate genetic diversity within sequences of different isolates, sequences of *pocmsp1*_*19*_ and *powmsp1*_*19*_ were aligned using GeneDoc version 2.7.0.

### Recombinant expression and purification of proteins

Genes including *pocmsp1*_*19*_ and *powmsp1*_*19*_ were subcloned into the pGEX-6p-1 expression vector which contained a glutathione S-transferase (GST)-tag fusion protein. Recombinant pGEX-6p-1^*pomsp119*^ were transferred into *Escherichia coli* strain BL21 pLysS cells to express PoMSP1_19_ proteins. Colonies of recombinants were cultured in Luria Bertani broth supplemented with ampicillin 50 µg/ml by shaking at 250 r.p.m. at 37 °C until the optical density (OD) at 600 nm reached 0.6–0.8. To induce the expression of recombinant PoMSP1_19_ (rPoMSP1_19_) proteins, isopropyl β-D-1-thiogalactopyranoside (0.1 mM) (TransGen Biotech, Beijing, China) was added and the culture was allowed to grow for another eight hours. Proteins were purified by Tanlen-bio Scientific (Wuxi, China). The rPoMSP1_19_-GST proteins were separated using 10% sodium dodecyl sulfate–polyacrylamide gel electrophoresis (SDS–PAGE) and detected via western blot and Coomassie brilliant blue staining (Beyotime Biotech, China). For western blot analysis, the proteins were electrophoresed on a polyvinylidene difluoride (PVDF) membrane (Immobilon; Millipore Sigma). Nonspecific bindings were blocked by incubation with 5% skimmed milk in Tris-buffered saline supplemented with 0.1% Tween-20 (TBST) at room temperature for 2 h. The membranes were incubated with anti-GST rabbit monoclonal antibody (CWBio Biotech) as the primary antibody at 1:2000 dilution overnight at 4 °C, and then washed three times with 0.1% TBST. Horseradish peroxidase (HRP)-conjugated goat anti-rabbit immunoglobulin G (IgG) (CWBio Biotech) was used as the secondary antibody for detection (at 1:5000 dilution for 1 h). Finally, the membranes were visualized using the ChemiDoc MP imaging system (Bio-Rad).

### Immunization of mice

Female BALB/c mice (Cavens; Changzhou, China) aged 6–8 weeks were intraperitoneally injected with 50 μg of rPocMSP1_19_-GST, rPowMSP1_19_-GST, GST, or phosphate-buffered saline (PBS) mixed with Freund’s complete adjuvant (Sigma, San Francisco, CA) as the primary immunization. The same amount of recombinant protein was mixed with incomplete Freund’s adjuvant then injected at days 21 and 42 after the initial injection to boost immunization. Mouse serum samples were collected and stored at − 80 ℃ on days 0, 7, 14, 28, 35, and 49 after the initial injection.

Western blot analysis was performed to detect antibodies directed against rPoMSP1_19_-GST from the sera of immunized mice. Concretely, rPoMSP1_19_–GST and GST proteins were transferred from SDS-PAGE onto PVDF membranes. The membranes were incubated with the sera of mice immunized with rPoMSP1_19_-GST as the primary antibody, with sera of the GST immunized group, or with the sera of those injected with PBS, the negative control group, and then incubated with HRP-conjugated goat anti-mouse IgG (Cowin Biotech) at 1:5000 dilution for detection.

### Enzyme-linked immunosorbent assay

Levels of IgG antibodies against rPoMSP1_19_-GST and GST in the sera of immunized mice were evaluated through enzyme-linked immunosorbent assay (ELISA) as previously described [35, 38]. For the assays, 50 ng of rPoMSP1_19_-GST or GST was immobilized on 96-well plates overnight at 4 °C in coating buffer (15 mM sodium carbonate and 35 mM sodium bicarbonate in distilled water) and blocked with 5% (weight/volume) non-fat milk in TBST at room temperature for 2 h. A 100-µl volume of a twofold serial dilution (1:10,000 to 1:5,120,000) of anti-rPoMSP1_19_-GST or anti-GST mouse sera was added to each well and incubated for 1 h at room temperature. The plates were washed three times with PBS containing 0.1% of Tween-20 (PBST) then incubated with HRP-conjugated goat anti-mouse IgG antibodies (Southern Biotech) at 1:5000 dilution for 1 h at room temperature. Finally, the plates were washed again and incubated with 100 µl of 3,3’,5,5’-tetramethylbenzidine (Invitrogen) substrate for 8 min in the dark, and the reaction was stopped with 50 µl of 2 M H_2_SO_4_ in each well. The absorbance was read at 450 nm. All samples were tested in duplicate and the mean absorbance was calculated.

The affinity test of the anti-rPoMSP1_19_-GST IgG antibody was performed in accordance with the above-mentioned ELISA test with the exception that the test here was repeated in a 96-well plate coated with the same recombinant proteins. More specifically, sera were incubated for 90 min at room temperature, following by washing of one of the plates with PBST while the other plate was first incubated with 100 µl of TBST containing 6 M urea for 10 min at room temperature before final washing with PBST. Finally, all the plates were incubated with HRP-conjugated goat anti-mouse IgG antibodies at 1:5000 dilution for 1 h at room temperature. The reaction was stopped with 50 µl of 2 M H_2_SO_4_ in each well and absorbance was measured at 450 nm. The avidity index (AI) was calculated as follows [39]:

$${\text{AI}}\, = \,\left( {{\text{OD45}}0{\text{ of a sample treated with 6}} {\text{Murea}}/{\text{OD45}}0{\text{ of a sample not treated with 6}} {\text{Murea}}} \right)\, \times \,{1}00$$

### Lymphocyte proliferation assays

Assays for lymphocyte proliferation were performed using Cell Counting Kit-8 (CCK-8; Beyotime Biotech), as previously described [35, 40]. Briefly, lymphocytes from mice immunized with rPoMSP1_19_-GST, GST and PBS (5 × 10^5^ cells/well) were treated with 10 µL of rPocMSP1_19_-GST (5 µg/mL), 10 µL of rPowMSP1_19_-GST (5 µg/mL), 10 µL of GST (5 µg/mL) or 10 µL of concanavalin A (ConA; 2 μg/mL) as the positive control in 96-well flat-bottomed microtiter plates then incubated for 72 h at 37 °C with 5% CO_2_. A 10-µl volume of CCK-8 was added to each well and the plates were incubated at 37 °C for 2 h. Finally, cell proliferation was measured at 450 nm by a microplate reader.

### Screening of serum samples

Serum samples from 29 cases of *P. ovale*-infected and 20 healthy individuals were screened by well-type amine arrays. The screening was performed as previously described [41, 42]. Briefly, modified glass slides (75 × 25 mm,) were prepared for the protein arrays (CapitalBio, Beijing, China) and warmed to room temperature before use. Teflon tapes with holes were pasted on array glass slides to ensure integrity of the array. One microliter of rPocMSP1_19_-GST, rPowMSP1_19_-GST and GST solution in PBS (100 ng/µl) was spotted into each well of the arrays and incubated for 2 h at 37 °C. Array slides were washed three times with PBST for 10 min and were blocked with 5% of bovine serum albumin in PBST at 37 °C for 2 h. The arrays were washed again and probed with 1 µl of serum samples at 1:200 dilution. Finally, 1 µl of Alexa Fluor 546 goat anti-human IgG (10 ng/µl; Invitrogen) in PBST was added to the arrays for antibody detection. The intensity of the serological responses was measured by a fluorescent microarray scanner (CapitalBio). The positive cut-off value was calculated as the mean fluorescence intensity (MFI) of negative controls plus 2 SD. A Mann–Whitney* U*-test was performed to compare differences in MFI between groups.

### Statistical analysis

GraphPad Prism software (version 5.0; GraphPad Software) was used for the statistical analyses and graphing. An unpaired two-tailed Student* t*-test was used for the comparison of mean values between samples; differences were considered statistically significant when *P*-values < 0.05.

## Results

### Genetic description and PCR analysis of ***pomsp1***_***19***_

**Fig. 1:**
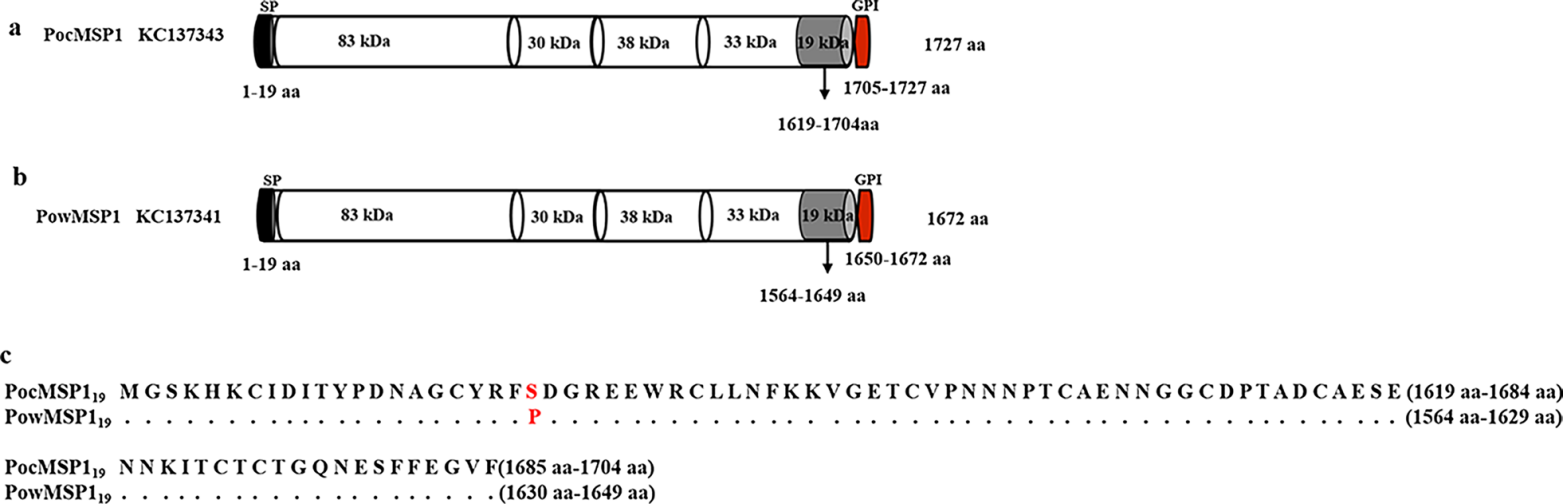
**a**, **b** Schematic diagram of protein structure and alignment of the C-terminal 19-kDa region of *Plasmodium ovale* merozoite surface protein 1 (*PoMSP1*_19_) amino acid (*aa*) sequences. **a**, **b** Signal peptide (*SP*;* black*), glycophosphatidylinositol (*GPI*; red), and expression area (*gray*) of proteins. **c** Alignment of amino acid sequences of *Plasmodium ovale curtisi *(Poc) MSP1_19_ (*PocMSP1*_*19*_) and *Plasmodium ovale wallikeri* (Pow) MSP1_19_ (*PowMSP1*_*19*_)

**Fig. 2:**
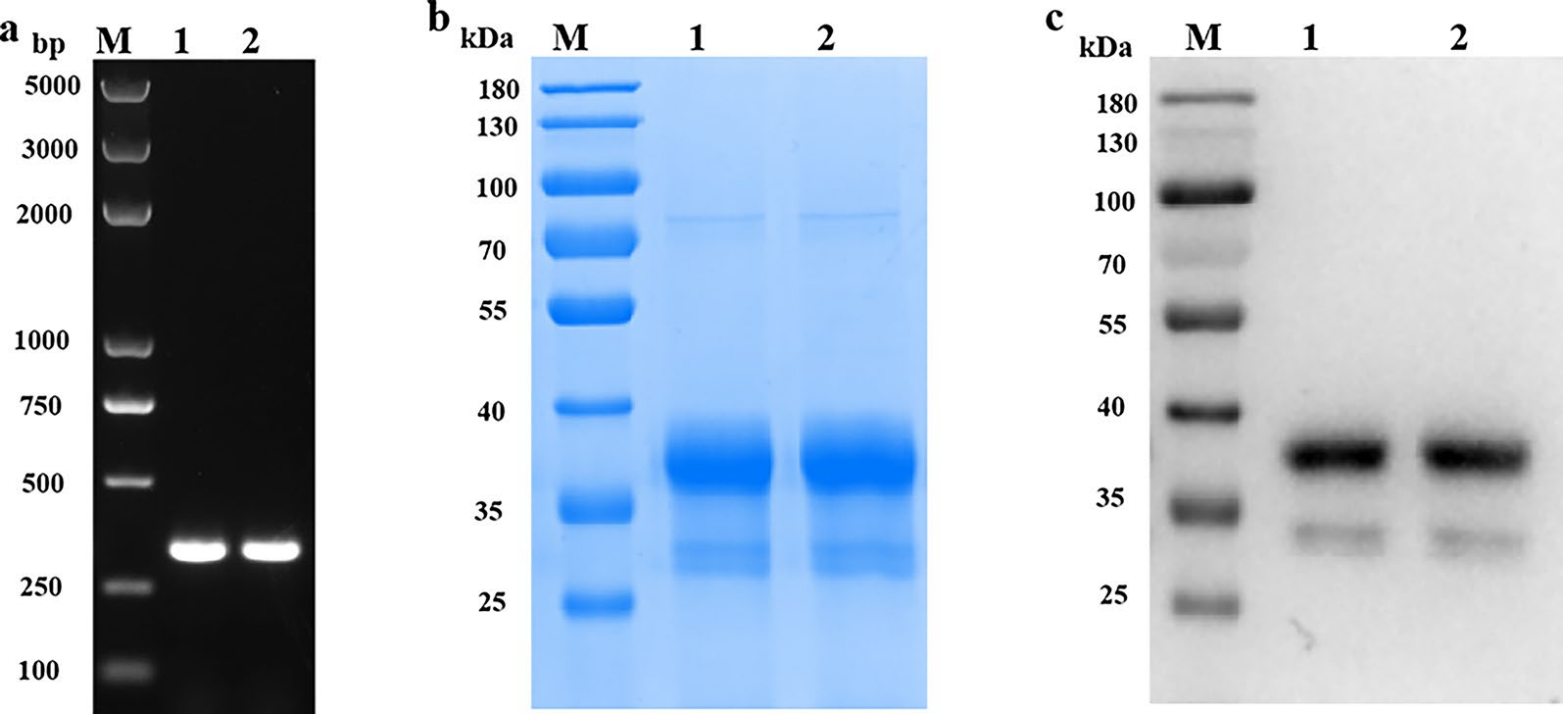
**a**, **b** Polymerase chain reaction (PCR) amplification of *pomsp1*_*19*_, and expression and purification of recombinant PoMSP1_19_ (rPoMSP1_19_)-glutathione S-transferase (GST) proteins. **a** The *pocmsp1*_*19*_ and *powmsp1*_*19*_ genes were amplified by PCR. **b** Purified rPoMSP1_19_-GST (~ 37 kDa) was resolved by 10% sodium dodecyl sulfate–polyacrylamide gel electrophoresis. **c** Western blot analysis of rPoMSP1_19_-GST using an anti-GST rabbit monoclonal antibody. *1* Purified rPocMSP1_19_-GST protein, *2* purified rPowMSP1_19_-GST protein, *M* marker; for other abbreviations, see Fig. 1

The full lengths of PocMSP1 (KC137343) and PowMSP1 (KC137341) were predicted to be 1727 and 1672 amino acids, respectively [34]. Following the proteolytic cleavage of MSP1, schematic diagrams of PocMSP1 and PowMSP1 were divided into seven domains as follows: signal peptide, 83-kDa domain, 30-kDa domain, 38-kDa domain, 33-kDa domain, 19-kDa domain, and glycolphosphatidylinositol (GPI) (Fig. 1a, b). Sequence alignment of amino acids between PocMSP1_19_ and PowMSP1_19_ showed only one amino acid difference for each (amino acids located at positions 1640 and 1585 in PocMSP1 and PowMSP1, respectively) (Fig. 1c). Clinical *P. ovale curtisi* and *P. ovale wallikeri* isolates (*n* = 20 and *n* = 17, respectively) that were used as sources of genomic DNA for PCR amplification are listed in Additional file 1: Table S1. PCR products of *pomsp1*_*19*_ genes were successfully amplified and showed a single 258-bp band on electropherograms (Fig. 2a). Alignment of *pomsp1*_*19*_ sequences from all isolates showed no amino acid mutation (Additional file 2: Fig S1), suggesting that *pomsp1*_*19*_ was completely conserved across the isolates.

### Expression, purification, and analysis of rPoMSP1_19_-GST proteins

The molecular weight of rPoMSP1_19_-GST was estimated as approximately 35 kDa (including the molecular weights of PocMSP1_19_/PowMSP1_19_ and pGEX-6p-1 expression vector containing a GST-tag fusion protein, estimated as 9 and 26 kDa, respectively). Recombinant proteins were efficiently expressed and purified, as shown in Fig. 2b, and presented in SDS-PAGE as a single band of approximately 37 kDa. Western blot analysis confirmed the expression of rPoMSP1_19_-GST proteins (Fig. 2c).

### Mice-derived antibodies against rPoMSP1_19_-GST recognized the recombinant proteins

**Fig. 3:**
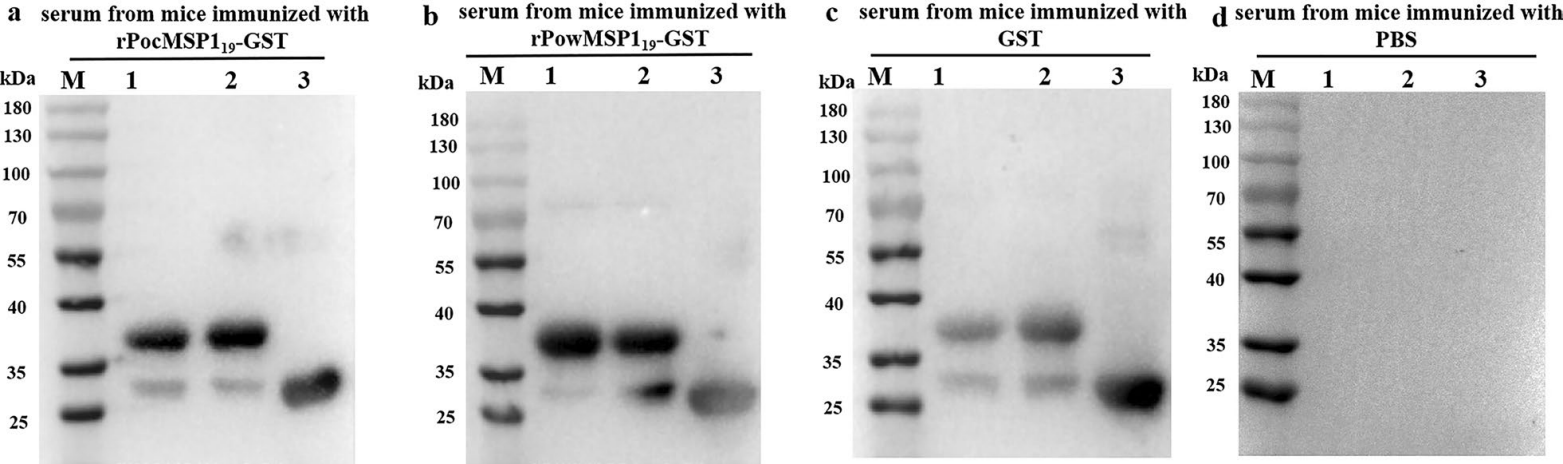
**a**–**d** Detection of antibodies in the sera of mice immunized with rPoMSP1_19_-GST. **a**–**c** Western blot analyses for the detection of rPoMSP1_19_-GST proteins by using antibodies from the sera of mice immunized with rPoMSP1_19_-GST and GST proteins. **d** Western blot analysis using antibodies from the sera of mice immunized with PBS. *1* Purified rPocMSP1_19_-GST protein, *2* purified rPowMSP1_19_-GST protein, *3* purified GST protein. For abbreviations, see Figs. 1 and 2

To verify whether anti-rPoMSP1_19_-GST antibodies were produced in the sera of immunized mice, we performed an immunoblot for a specific ~ 37-kDa band of purified rPoMSP1_19_-GST proteins. As expected, sera from mice immunized with rPocMSP1_19_-GST and rPowMSP1_19_-GST detected rPocMSP1_19_-GST and rPowMSP1_19_-GST, respectively (Fig. 3a, b). In addition, sera from mice immunized with GST could also recognize the recombinant proteins (Fig. 3c). No band was found for the recombinant proteins treated with sera from mice immunized with PBS (negative control). These results indicated that rPoMSP1_19_-GST could induce anti–rPoMSP1_19_-GST antibodies in mice.

Immunized mouse-generated anti-rPocMSP1_19_, anti-rPowMSP1_19_, and anti-GST sera also cross-reacted with the rPoMSP1_19_ proteins and with GST (Fig. 3a–c). Sera from mice immunized with rPocMSP1_19_-GST could recognize rPowMSP1_19_-GST and GST (Fig. 3a). Similarly, sera from mice immunized with rPowMSP1_19_-GST could also recognize rPocMSP1_19_-GST and GST (Fig. 3b). These results showed that anti–rPocMSP1_19_-GST and anti–rPowMSP1_19_-GST antibodies had the ability to cross-react with PocMSP1_19_ and PowMSP1_19_ antigens.

### Levels of immune responses in mice immunized with rPoMSP1_19_-GST

**Fig. 4:**
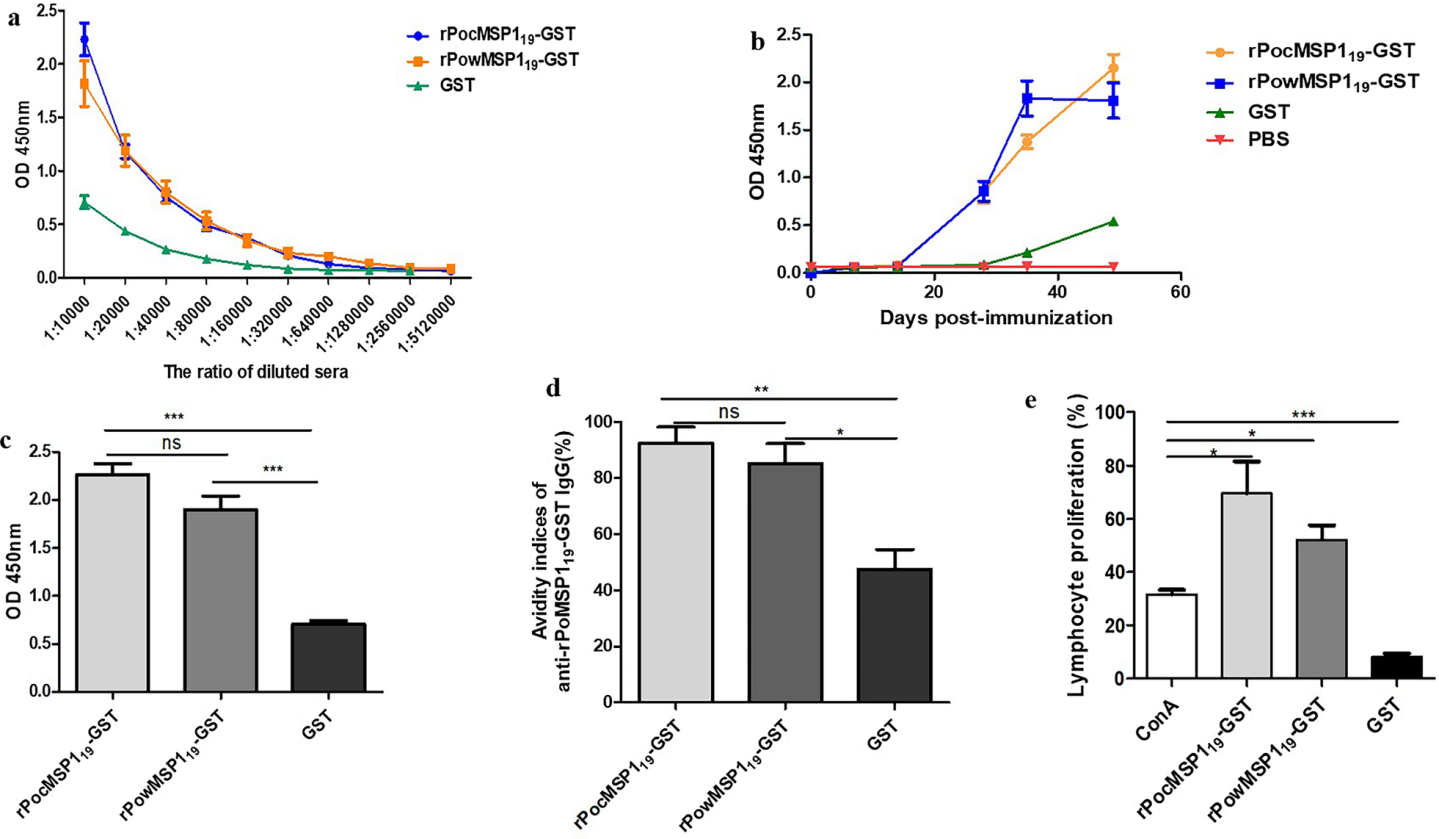
**a**–**e** Immune responses in mice immunized with rPoMSP1_19_-GST. **a** Sera from immunized mice were multiply diluted (from 1:10,000 to 1:5,120,000), and the data are expressed as average optical density (*OD*) values. **b** Increasing trend of immunoglobulin G (IgG) levels detected using ELISA. **c** Specific immune responses in sera from mice immunized with different proteins confirmed by testing the interaction between antibodies and rPocMSP1_19_-GST, rPowMSP1_19_-GST and GST. The mean OD shows the strength of specific immune responses. **d** Avidity indices of anti-rPoMSP1_19_-GST IgG determined by ELISA. **e** Splenocytes from immunized mice were stimulated with recombinant proteins, and concanavalin A (*ConA*) was used as a positive control. * *P* < 0.05, ** *P* < 0.01, *** *P* < 0.001; not significant (*ns*) *P* > 0.05. For other abbreviations, see Figs. 1 and 2

To measure levels of immune responses against rPoMSP1_19_-GST or PBS (negative control) in mice, ELISA was performed using rPoMSP1_19_-GST and GST proteins as the coating antigens. The results showed that both rPocMSP1_19_-GST and rPowMSP1_19_-GST were immunogenic (Fig. 4). The average antibody titers were determined by ELISA at 49 days after the first intraperitoneal injection. The proteins rPocMSP1_19_-GST and rPowMSP1_19_-GST induced comparable antibody responses, with end-point titers ranging from 1:10,000 to 1:2,560,000 (Fig. 4a). After three consecutive immunizations, high IgG antibody responses were induced in mice immunized with rPoMSP1_19_-GST (Fig. 4b, c). The titration curves for GST were indicative of low responses compared to those for rPoMSP1_19_-GST proteins. In addition, in comparison to immunization with rPoMSP1_19_-GST proteins, GST immunization failed to induce high IgG antibody responses in mice, and PBS induced no IgG antibody response at all. These results suggested that immunization of mice with rPoMSP1_19_-GST proteins induced higher levels of IgG antibodies (rPocMSP1_19_-GST, mean avidity, 92.57%, *P* = 0.0024; rPowMSP1_19_-GST, mean avidity, 85.32%, *P* = 0.014) than immunization of mice with GST (mean avidity, 37.84%) (Fig. 4d). However, there was no statistically significant difference in immune response between the rPocMSP1_19_-GST and rPowMSP1_19_-GST groups of mice (*P* > 0.05).

Cellular immune responses induced by rPoMSP1_19_-GST were assessed through spleen lymphocyte proliferation assays. The proliferation of splenocytes in vitro under rPocMSP1_19_-GST, rPowMSP1_19_-GST, GST, and ConA (positive control) stimulations was determined. Cell proliferation induced by rPocMSP1_19_-GST was 69.51% and that induced by rPowMSP1_19_-GST was 52.17%. Collectively, rPoMSP1_19_-GST proteins induced a stronger proliferation response of spleen cells (rPocMSP1_19_-GST, *P* = 0.0471; rPowMSP1_19_-GST, *P* = 0.026) than ConA. GST failed to induce cell proliferation (*P* < 0.00001; Fig. 4e).

### Cross‑reactivity of rPoMSP1_19_-GST proteins with anti‑rPoMSP1_19_-GST antibodies

**Fig. 5:**
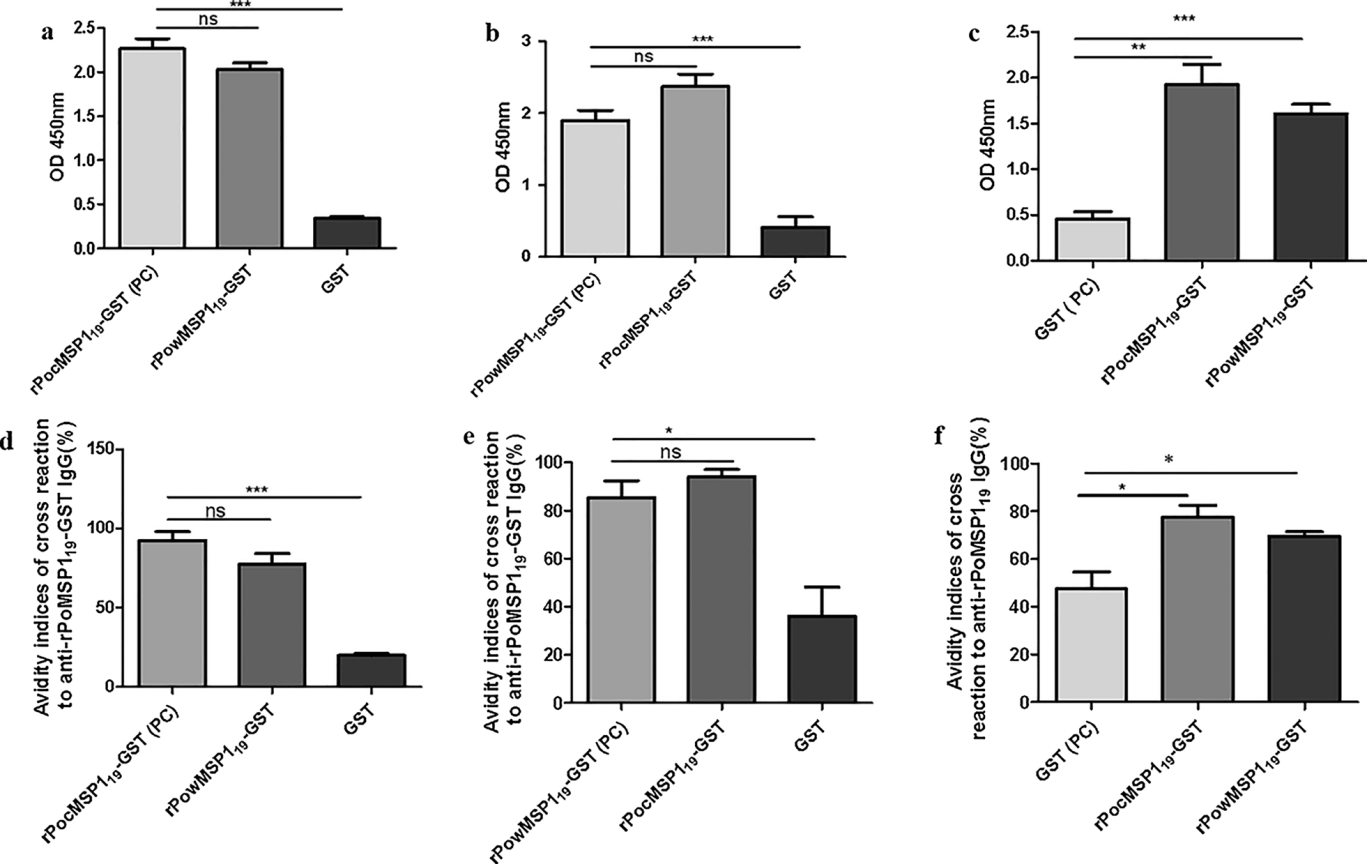
**a**–**e** Cross-reactivity of rPoMSP1_19_-GST proteins with antisera from mice immunized with rPoMSP1_19_-GST. **a** Cross-reactivity of rPowMSP1_19_-GST and GST with sera of mice immunized with rPocMSP1_19_-GST protein. **b** Cross-reactivity of rPocMSP1_19_-GST and GST with sera of mice immunized with rPowMSP1_19_-GST protein. **c** Cross-reactivity of rPoMSP1_19_-GST with sera from mice immunized with GST protein. **c**–**e** Avidity indices of cross-reaction to anti-rPoMSP1_19_-GST IgG [positive control (*PC*)]. The data are shown as the mean OD (the OD reflects the magnitude of the immune response). * *P* < 0.05, ** *P* < 0.01, *** *P* < 0.001; ns *P* > 0.05. For other abbreviations, see Figs. 1, 2 and  4

We examined cross-reactivity between rPocMSP1_19_-GST and rPowMSP1_19_-GST using ELISA. rPocMSP1_19_-GST could recognize IgG of sera from mice immunized with rPowMSP1_19_-GST (Fig. 5a, d), with no significant difference observed in cross-reactivity and avidity indices of cross-reaction (*P* > 0.05). However, anti-GST IgG antibodies showed a significantly lower level of cross-reactivity and avidity indices of cross-reaction with rPocMSP1_19_-GST than in reaction of rPocMSP1_19_-GST with anti-rPocMSP1_19_-GST IgG (*P* < 0.001; Fig. 5a, d). In addition, the cross-reactivity and affinity of anti-rPocMSP1_19_-GST antibodies and rPowMSP1_19_-GST showed no significance difference in comparison to the reaction of rPowMSP1_19_-GST with anti-rPowMSP1_19_-GST antibodies (Fig. 5b, e). Unexpectedly, rPocMSP1_19_-GST and rPowMSP1_19_-GST showed a significantly higher cross-reactivity and affinity than those of GST with anti-GST antibodies (*P* < 0.05) (Fig. 5c, f). These results suggested that either rPocMSP1_19_-GST or rPowMSP1_19_-GST had the ability to recognize and bind with high-affinity antibodies induced in mice immunized with rPoMSP1_19_-GST.

### Humoral immune responses to rPoMSP1_19_-GST in ***P. ovale*** infections

**Fig. 6:**
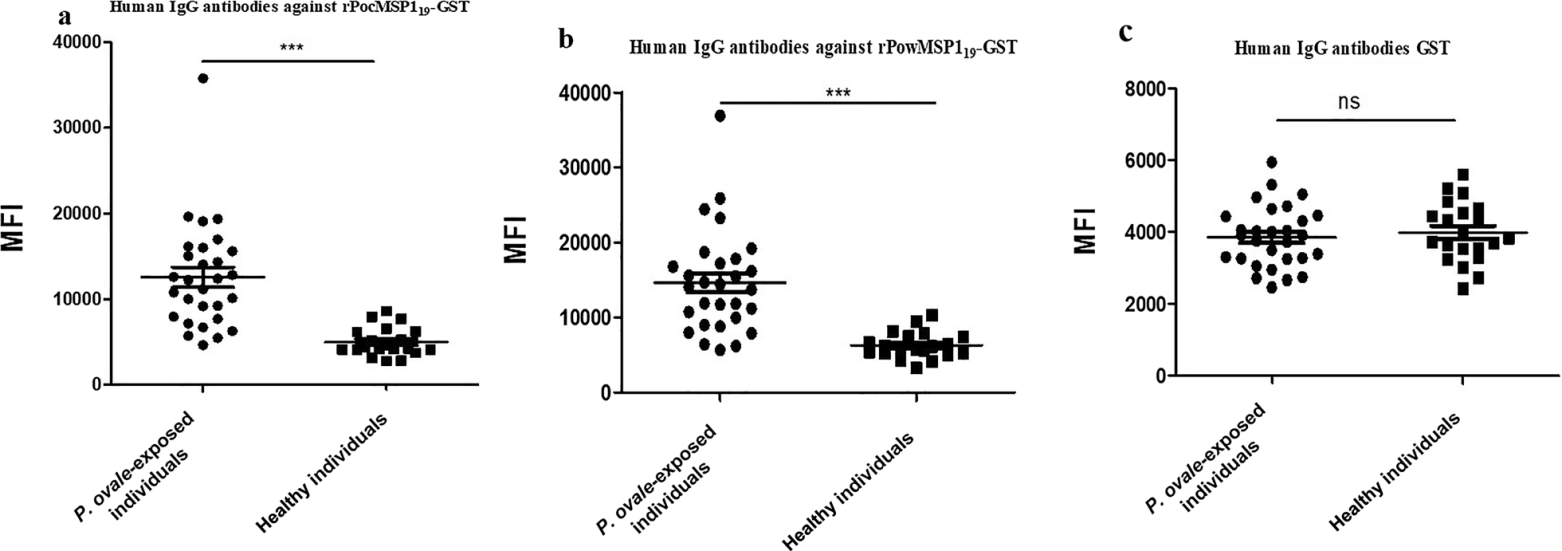
**a**–**c** Analysis of humoral immune responses to rPoMSP1_19_-GST in *Plasmodium ovale* infections. **a** IgG responses to rPocMSP1_19_-GST in patients infected with *P. ovale* and in healthy individuals. **b** IgG responses to rPowMSP1_19_-GST in patients infected with *P. ovale* and in healthy individuals. **c** IgG responses to GST in *P. ovale*-infected patients and in healthy individuals. * *P* < 0.05, ** *P* < 0.01, *** *P* < 0.001; ns *P* > 0.05. For abbreviations, see Figs. 1, 2 and  4

Naturally acquired humoral immune responses in patients infected with *P. ovale* were assessed against rPoMSP1_19_-GST. Protein microarrays were used to screen anti-rPoMSP1_19_-GST antibodies in 29 serum samples from *P. ovale*-infected individuals and 20 serum samples from healthy individuals (Additional file 3: Table S2). Patients infected with *P. ovale* showed significantly higher MFI of total IgG against rPocMSP1_19_-GST and rPowMSP1_19_-GST than healthy individuals (*P* < 0.0001) (Fig. 6a, b). Anti-rPocMSP1_19_-GST antibodies showed sensitivity and specificity of 89.96% (MFI value of 26/29 patient samples > cut-off value of 5752.4) and 75% (MFI value of 15/20 healthy samples < 5752.4), respectively. Anti-rPowMSP1_19_-GST antibodies showed sensitivity and specificity of 89.96% (MFI value of 26/29 patient samples > cut-off value 7092) and 75% (MFI value of 15/20 healthy samples < cut-off value 7092), respectively. However, no statistically significant difference was observed in the MFI of total IgG against GST protein between *P. ovale*-infected and healthy individuals (*P* = 0.5964; Fig. 6c). These results showed that both rPocMSP1_19_-GST and rPowMSP1_19_-GST are targets of signatures of exposure and immunity.

## Discussion

Molecules of blood-stage malaria parasites are components of interest for the development of effective malaria vaccines [43]. Several merozoite surface proteins are regarded as promising malaria vaccine candidates because they are targets of host immune selection and play essential roles in erythrocyte invasion [28, 44]. In the first rodent model used to study a malaria vaccine, MSP1 was proven to provide protection against challenge infection [45]. That study showed that after MSP1 undergoes proteolytic cleavage only MSP1_19_ is carried into the invaded erythrocytes. In a later study, antibodies against MSP1_19_ were shown to prevent merozoites from invading erythrocytes in vitro [20]. Antigenic diversity increases the difficulty of developing an effective malaria vaccine, as it helps parasites to escape from host immune responses [46]. In the present study, sequences of *pomsp1*_*19*_ were completely conserved in 20 *P. ovale curtisi* and 17 *P. ovale wallikeri* clinical isolates. These results are consistent with those from previous studies which reported genetic conservation of *msp1*_*19*_ across malaria parasites [33, 36, 37]. Immune-mediated selection pressure is an important mechanism as it may lead to antigenic diversity [47]. In the present study, we found that the C-terminal region of PoMSP1_19_ is under relatively lower selective pressure than the N-terminal of PoMSP1, which has also been shown to have low genetic diversity [34].

Antibodies play an important role in protection against clinical disease. Earlier studies suggested that antibody responses to PfMSP1_19_ might protect children from high levels of blood-stage parasitemia and clinical malaria [32]. In the present study, cross-reaction between rPocMSP1_19_-GST and rPowMSP1_19_-GST was detected. This cross-reaction indicates that rPocMSP1_19_ and rPowMSP1_19_ share similar antigenic determinants and, therefore, PoMSP1_19_ might possess species-specific efficacy as a vaccine candidate. In addition, as rPoMSP1_19_-GST protein contains the GST tag, rPocMSP1_19_-GST and rPowMSP1_19_-GST could be detected by using the sera of mice immunized with GST protein as a non-specific control. Of the five classes of immunoglobulins, IgG is well-known as one that plays a critical role in malaria immunity [48]. A significantly higher level of IgG antibodies was detected in the sera of mice immunized with rPoMSP1_19_-GST proteins than in the sera of the control group of mice immunized with GST protein (Fig. 4), which showed that rPoMSP1_19_-GST induced immune responses in mice. There was no significant difference in cross-reactivity between anti-rPocMSP1_19_-GST IgG and anti-rPowMSP1_19_-GST IgG antibodies, while the affinity index of anti-rPoMSP1_19_-GST IgG was higher than that of the GST group in the cross-reactivity and autoantigen binding test (Fig. 5). These findings imply that antibodies induced in mice immunized with rPoMSP1_19_-GST can bind tightly to antigens and tend to mature. Although anti-GST antibodies were induced in mice immunized with GST protein, affinity maturation was not promoted. High-affinity antibodies are advantageous in a number of biological functions [49]. A better understanding of cellular responses can help in the development of effective blood-stage vaccine candidates for malaria. Lymphocytes play a key role in the immune system and have been shown to be specific for immune responses to infectious microorganisms and other foreign substances [50]. Lymphocyte proliferation assays are widely used to determine T cell immune responsiveness to an antigenic stimulus. The current study showed that rPocMSP1_19_-GST could stimulate the proliferation of T cells (Fig. 4f). This result is particularly instructive as it proves that rPocMSP1_19_-GST can induce cellular immune responses in mice, and more importantly, proves the immunogenicity of rPoMSP1_19_-GST in mice.

A number of studies have reported serologic analyses that investigated IgG antibodies against specific antigens of *P. falciparum* and *P. vivax* to assess infection incidence and immunity levels [17, 51, 52]; however, the question of response specificity has not been fully explored. In this study, we also analyzed humoral immune responses in *P. ovale* infections. Most of the *P. ovale*-infected serum samples showed positive antibody responses to rPoMSP1_19_-GST, with a high sensitivity (89.96%) and specificity (75%). The differences in immune responses were not related to the presence of GST protein as no significant difference was observed in the MFI of total IgG against GST protein between *P. ovale*-infected and healthy individuals (Fig. 6). These data confirmed the antigenicity of rPoMSP1_19_-GST, which may indirectly reflect or contribute to protection against* P. ovale* malaria infection as humoral immune responses are partly involved in preventing clinical malaria [53]. Seroepidemiological studies have been particularly informative in areas of low transmission where the sensitivity of surveys for prevalence of malaria parasites is impacted by the low number of parasite-infected individuals [54]. Previous studies have used PfMSP1_19_ and PvMSP1_19_ as serological markers to detect the prevalence of malaria parasites [55, 56]. Due to the limited number of serum samples in the present study we were not able to compare differences in the signal of serological responses with respect to other criteria such as gender and parasite density. However, PoMSP1_19_ as a biomarker of exposure is worthy of further study.

## Conclusions

This study demonstrated that *pomsp1*_*19*_ sequences were completely conserved in clinical *P. ovale curtisi* and *P. ovale wallikeri* isolates. Furthermore, the immunogenicity of rPoMSP1_19_ in mice was comprehensively analyzed. The informative results presented here contribute to the knowledge base for the development of a PoMSP1_19_-based vaccine. In addition, the naturally acquired antibody responses against rPoMSP1_19_ shown here indicate that further study of PoMSP1_19_ could provide new insights for serological research. The cross-reactivity of PoMSP1_19_ with sera from patients infected with *P. falciparum* or *P. vivax* needs to be explored in future studies, as does the clinical protection against malaria afforded by anti-PoMSP1_19_ antibodies.

## Supplementary Information

**Additional file 1: Table S1.** Information on imported *Plasmodium ovale curtisi* and *Plasmodium ovale wallikeri* isolates used in this study.

**Additional file 2: Figure S1.** Alignments of amino acid sequences of *Plasmodium ovale curtisi* and *Plasmodium ovale wallikeri* MSP1_19_ in all amplified clinical isolates.

**Additional file 3: Table S2.** Information on serum samples from patients infected with *Plasmodium ovale* used for the microarrays.

## Data Availability

The data supporting the conclusions of this article are included within the article and its additional files. The new sequences identified in this study have been deposited in GenBank with the accession numbers MZ766552-MZ766553.

## Abbreviations

ELISA: Enzyme-linked immunosorbent assay
GPI: Glycophosphatidylinositol
GST: Glutathione S-transferase
IgG: Immunoglobulin G
MSP1: Merozoite surface protein 1
PCR: Polymerase chain reaction
SDS-PAGE: Sodium dodecyl sulfate–polyacrylamide gel electrophoresis
