# Supplementary material for: Genetic diversity and immunogenicity of the merozoite surface protein 1 C-terminal 19-kDa fragment of Plasmodium ovale imported from Africa into China
Source: Parasit Vectors. 2021 Nov 24;14:583. doi: 10.1186/s13071-021-05086-6 (PMC8611641; doi:10.1186/s13071-021-05086-6)
Supplement: Supplementary file 1 — Additional file 1: Table S1. Information on imported Plasmodium ovale curtisi and Plasmodium ovale wallikeri isolates used in this study. [file 13071_2021_5086_MOESM1_ESM.docx]

| **Table S1 Information on imported P. ovale curtisi and P. ovale wallikeri isolates** | | | |
| --- | --- | --- | --- |
| **Species confirmation** | **Isolate number** | **Country of Origin** | **Parasitaemia level** |
| *P. ovale curtisi* | Poc-14 | Angola | 5922 |
|  | Poc-16 | Equatorial Guinea | 916 |
|  | Poc-18 | Equatorial Guinea | 736 |
|  | Poc-20 | Equatorial Guinea | 3124 |
|  | Poc-21 | Equatorial Guinea | 3477 |
|  | Poc-26 | Equatorial Guinea | 1950 |
|  | Poc-35 | Equatorial Guinea | 1500 |
|  | Poc-36 | Equatorial Guinea | 1800 |
|  | Poc-37 | Equatorial Guinea | 653 |
|  | Poc-39 | Equatorial Guinea | 568 |
|  | Poc-44 | Equatorial Guinea | 1052 |
|  | Poc-52 | Congo-Kinshasa | 3830 |
|  | Poc-60 | Gabon | 9363 |
|  | Poc-62 | Cameroon | 2736 |
|  | Poc-71 | Niger | 2500 |
|  | Poc-74 | Nigeria | 440 |
|  | Poc-75 | Nigeria | 2219 |
|  | Poc-77 | Nigeria | 1013 |
|  | Poc-83 | Nigeria | 1200 |
|  | Poc-84 | Nigeria | 2629 |
| *P. ovale wallikeri* | Pow-6 | Angola | 3354 |
|  | Pow-12 | Angola | 5876 |
|  | Pow-23 | Equatorial Guinea | 9339 |
|  | Pow-30 | Equatorial Guinea | 4168 |
|  | Pow-40 | Equatorial Guinea | 6200 |
|  | Pow-41 | Equatorial Guinea | 4277 |
|  | Pow-45 | Equatorial Guinea | 6500 |
|  | Pow-47 | Equatorial Guinea | 285 |
|  | Pow-54 | Republic of Congo | 32592 |
|  | Pow-56 | Republic of Congo | 208000 |
|  | Pow-59 | Republic of Congo | 1800 |
|  | Pow-63 | Republic of Congo | 3289 |
|  | Pow-67 | Gabo | 10370 |
|  | Pow-74 | Republic of Liberia | 8380 |
|  | Pow-77 | Mozambique | 483 |
|  | Pow-85 | Nigeria | 1448 |
|  | Pow-89 | The Republic of Sierra Leone | 6151 |
