# Supplementary material for: Genetic diversity and immunogenicity of the merozoite surface protein 1 C-terminal 19-kDa fragment of Plasmodium ovale imported from Africa into China
Source: Parasit Vectors. 2021 Nov 24;14:583. doi: 10.1186/s13071-021-05086-6 (PMC8611641; doi:10.1186/s13071-021-05086-6)
Supplement: Supplementary file 3 — Additional file 3: Table S2. Information on serum samples from patients infected with Plasmodium ovale used for the microarrays. [file 13071_2021_5086_MOESM3_ESM.docx]

| **Table S2 Information on serum samples from patients infected with *Plasmodium ovale* used for microarrays** | | | |
| --- | --- | --- | --- |
| **Serum number** | **Age** | **Country of Origin** | **Parasitaemia level** |
| JSPO01 | 31 | Equatorial Guinea | 1052 |
| JSPO02 | 47 | Angola | 308 |
| JSPO03 | 33 | Republic of Malawi | 4302 |
| JSPO04 | 27 | Nigeria | 2635 |
| JSPO05 | 46 | Equatorial Guinea | 8432 |
| JSPO06 | 31 | Gabo | 2016 |
| JSPO07 | 49 | Ghana | 11851 |
| JSPO08 | 33 | Côte d'Ivoire | 629 |
| JSPO09 | 33 | Côte d'Ivoire | 629 |
| JSPO10 | 49 | Ghana | 11851 |
| JSPO11 | 46 | Centrafricaine | 7824 |
| JSPO12 | 44 | Nigeria | 4347 |
| JSPO13 | 48 | Republic of Congo | 2597 |
| JSPO14 | 58 | Angola | 17891 |
| JSPO15 | 51 | Nigeria | 5285 |
| JSPO16 | 42 | Nigeria | 9600 |
| JSPO17 | 51 | Angola | 9841 |
| JSPO18 | 53 | Algérie | 1140 |
| JSPO19 | 30 | Nigeria | 4845 |
| JSPO20 | 46 | Nigeria | 3200 |
| JSPO21 | 38 | Angola | 1310 |
| JSPO22 | 52 | Equatorial Guinea | 2370 |
| JSPO23 | 54 | Equatorial Guinea | 622 |
| JSPO24 | 43 | Republic of Cameroon | 2526 |
| JSPO25 | 42 | Republic of Congo | 4800 |
| JSPO26 | 44 | Equatorial Guinea | 1751 |
| JSPO27 | 44 | Equatorial Guinea | 1751 |
| JSPO28 | 34 | Angola | 22118 |
| JSPO29 | 48 | Angola | 5224 |
